# Modulation of the Antibiotic Activity by Extracts from *Amburana cearensis* A. C. Smith and *Anadenanthera macrocarpa* (Benth.) Brenan

**DOI:** 10.1155/2013/640682

**Published:** 2012-12-27

**Authors:** Fernando G. Figueredo, Emerson O. Ferreira, Bruno F. F. Lucena, Cícero M. G. Torres, Daniel L. Lucetti, Elaine C. P. Lucetti, João Marcos F. L. Silva, Francisco A. V. Santos, Cássio R. Medeiros, Gardênia M. M. Oliveira, Aracélio V. Colares, José G. M. Costa, Henrique D. M. Coutinho, Irwin R. A. Menezes, Júlio C. F. Silva, Marta R. Kerntopf, Patrícia R. L. Figueiredo, Edinardo F. F. Matias

**Affiliations:** ^1^Faculdade Leão Sampaio, 63100-000, Juazeiro do Norte, CE, Brazil; ^2^Faculdade de Medicina Estácio de Juazeiro do Norte, 63100-000, Juazerio de Norte, CE, Brazil; ^3^Laboratório de Pesquisa em Produtos Naturais, Universidade Regional do Cariri, 63105-000, Crato, CE, Brazil; ^4^Laboratório de Microbiologia e Biologia Molecular, Universidade Regional do Cariri, 63105-000, Crato, CE, Brazil; ^5^Departamento de Química Biológica, Universidade Regional do Cariri, 63105-000, Crato, CE, Brazil; ^6^Laboratório de Farmacologia e Química Molecular, Universidade Regional do Cariri, 63105-000, Crato, CE, Brazil

## Abstract

The aim of this study was to verify the possible interactions between ethanol extracts of *Amburana cearensis* A. C. Smith and *Anadenanthera macrocarpa* (Benth.) Brenan, combined with six antimicrobial drugs against multiresistant strains of *Staphylococcus aureus* and *Escherichia coli* isolated from humans. The antibacterial activity of the extracts was determined using the minimum inhibitory concentration (MIC). The microdilution assay was performed to verify the interactions between the natural products and the antibiotics using a subinhibitory concentration. The activity of amikacin associated with the extract of *Anadenanthera macrocarpa* against EC 27 was enhanced, demonstrating an MIC reduction from 128 to 4 **μ**g/mL. Among the **β**-lactams, no potentiation on its activity was observed, with exception to the antagonism of the natural products with ampicillin against *S. aureus* 358.

## 1. Introduction

The research for new antibacterial substances becomes necessary due to increase of the antibiotic resistance of clinically important pathogens [1]. Due to this fact, substances derived from plants could be attractive alternatives [2, 3]. Natural products from plant may change or modulate the action of the antibiotic, enhancing or reducing the activity of this drug [4]. In recent years, many plants have been evaluated not only for the direct antibacterial action, but also as a modulator of the antibiotic activity [5, 6].

The use of natural products, mainly the chemical components of plants with antimicrobial properties have contributed to significant results in therapeutic treatments [7–9].

The *Amburana cearensis* (German) A. C. Smith, Fabaceae, is a tree which reaches 10–12 m of height [10]. Also known as “Cumaru”, it has been explored for use in fine furniture making, sculpture and carpentry, being listed as a threatened species [11]. Moreover, due to their medicinal properties, the bark and seeds are used to produce popular drugs for the treatment of cough, asthma, bronchitis, and pertussis. The species is still used in the perfume industry [12]. Medical trials have demonstrated preclinical anti-inflammatory, bronchodilator and analgesic activity for the hydroalcoholic extract, being possible to associate these effects of coumarin and flavonoidic fraction [13, 14].

The *Anadenanthera macrocarpa* is a species belonging to Mimosoideae [15]. This is a species of “angico” with larger geographic areas, occurring from southern Bolivia to northern Argentina, in Brazil, and is not only found in southern region [16]. Popular medicine has been used against several diseases through the preparation of syrups and lickers, it is used for the treatment of coughs, bronchitis, fads, external wounds and inflammation [17].

The objective of this study was to realize the phytochemical prospecting and assay of the *in vitro* ethanolic extracts of leaves of *A. Cearensis* and *A. macrocarpa* to determine the antibacterial activity and the modifying antibiotic activity of aminoglycosides and beta-lactams against the *Escherichia coli* and *Staphylococcus aureus*.

## 2. Material and Methods

### 2.1. Bacterial Material

The bacterial strains used were *E. coli* (EC-ATCC10536 and EC27) and *S. aureus* (SA-ATCC25923 e SA358) with a resistance profile identified in Table 1. All strains were maintained on heart infusion agar (HIA, Difco Laboratories Ltd.). Before the tests, the strains were grown for 18 h at 37°C in broth brain heart infusion (BHI, Difco Laboratories Ltd.).

### 2.2. Plant Material

Leaves of *Amburana cearensis* and *Anadenanthera macrocarpa* were collected at Penaforte, Ceara, Brazil. The plant material was identified and a voucher specimen was placed in the respective herbal collections (Table 2).

### 2.3. Preparation of Ethanol Extracts of Amburana Cearensis and Anadenanthera Macrocarpa

 For the preparation of extracts, leaves were collected and weighed (Table 3). The material was powdered and wrapped in a container with an amount of solvent to submerge the plant material by 72 hours. After this time, the eluent was filtered and concentrated in a rotary vacuum condenser (model Q-344B-Quimis, Brazil) and in an ultrathermal bath (model Q-214 M2-Quimis, Brazil) [18]. For the tests, the solutions used were prepared from extracts in a concentration of 10 mg/mL dissolved in DMSO (dimethyl-sulfoxide), then diluted with distilled water to a concentration of 1024 *μ*g/mL.

### 2.4. Phytochemical Prospecting

The phytochemicals tests to detect the presence of heterosides, tannins, flavonoids, steroids, triterpenes, coumarins, quinones, organic acids, and alkaloids were performed according to the method described by Matos [19]. The tests were based on visual observation of the change in color or formation of precipitate after the addition of specific reagents.

### 2.5. Antibacterial Activity Test

The MIC (minimal inhibitory concentration) was determined in a microdilution assay utilizing an inoculum of 100 *μ*L of each strain, suspended in brain heart infusion (BHI) broth up to a final concentration of 10^5^ CFU/mL in 96-well microtiter plates, using twofold serial dilutions. Each well received 100 *μ*L of each extract solution. The final concentrations of the extracts varied from 512 to 8 *μ*g/mL. MICs were recorded as the lowest concentrations required to inhibit growth. The minimal inhibitory concentration for the antibiotics was determined in BHI by the microdilution assay utilizing suspensions of 10^5^ CFU/mL and a drug concentration range from 2.5 to 0.0012 mg/mL (twofold serial dilutions). MIC was defined as the lowest concentration at which no growth was observed. For the evaluation of the extracts as modulators of the resistance to the antibiotics, MIC of the antibiotics was determined in the presence or absence of EEAC and EEAM at subinhibitory concentrations (8 *μ*g/mL) and the plates were incubated for 24 h at 37°C. Each antibacterial assay for MIC determination was carried out in triplicate.

### 2.6. Evaluation of the Modulation of Extracts on the Resistance to Aminoglycosides and *β*-Lactams Antibiotics

 To evaluate the extracts as modulators of antibiotic action, the MICs of antibiotics of the class aminoglycoside and beta-lactams, were evaluated in the presence and absence of the extracts in sterile microplates. The antibiotics were evaluated at concentrations ranging from 512 to 0.5 mg/mL. All antibiotics tested were obtained from Sigma. The extracts were mixed in BHI broth at 10% sub—inhibitory concentrations obtained and determined after the test evaluation of MIC, and for the modulation test concentration was used a concentration of extract referring the MIC diluted 8 times (MIC/8). The preparation of the antibiotic solutions was performed by adding sterile distilled water in a double concentration (1024 *μ*g/mL) in relation to the initial concentration set volume of 100 *μ*L and serially diluted 1 : 1 in 10% BHI broth. In each well with 100 *μ*L of culture medium containing the bacterial suspension diluted (1 : 10). The same controls used in the evaluation of MIC for the extracts were used for the modulation [4]. The plates were filled and incubated at 35°C for 24 hours, and after that the reading was evidenced by the use of resazurin as previously mentioned in the test of determination of the MIC.

## 3. Results and Discussion

The extracts evaluated in this work showed the yields demonstrated in Table 3, where we observed a higher yield of extract *A. macrocarpa* compared to *A. cearensis*. To perform the microdilution assay, the extracts were diluted in DMSO obtaining a solution of concentration of 10 mg/mL. A pilot study was conducted using only DMSO, but no antibacterial or modulatory activity was observed, indicating nontoxic effect.

Table 4 shows the presence of various potentially bioactive compounds in the extracts evaluated, like phenols, tannin pyrogallatos, tannin phlobaphenes, anthocyanins, anthocyanidins, flavones, flavonols, xanthones, chalcones, aurones, flavononols, leucoanthocyanidins, catechins, flavonones, and alkaloids. Through phytochemical prospecting of extracts, it was possible to identify the presence of several classes of secondary metabolites that exhibit a wide variety of biological activities such as antimicrobial [20–22], antioxidant [23], antitumor and antiophidic [24].

Table 5 shows the determination of minimum inhibitory concentration (MIC) of ethanol extracts tested against *E. coli* and *S. aureus* of reference and multiresistant. Comparatively, the extracts EEAM and EEAC showed the same MIC with the exception of EEAC against SA-ATCC 25923 which showed a better MIC of 512 *μ*g/mL.

Several medicinal plants were used as a source of many antimicrobial drugs used in the treatment of infectious diseases, including against bacteria multiresistant to antibiotics [25]. It is known that the synergistic action of the natural products with antimicrobial agents is commonly used in the therapeutic treatment [26, 27]. 

Table 6 shows the interference of the extracts on the activity of aminoglycosides, demonstrating a modulation in the activity of antibiotics, reducing the MICs. The more representative effect was observed with the association of EEAM and amikacin, an increase being observed in the antibiotic activity against EC27, reducing the MIC of the antibiotic from 128 to 4 *μ*g/mL.

Due to absorption into the intracellular space, the cell toxicity is common to all aminoglycosides (except to streptomycin). Nephrotoxicity, ototoxicity, and neuromuscular blockade are the most important toxic effects of aminoglycosides [7, 28]. The reported frequency of these side effects is highly variable due to different criteria used for diagnosis [29]. The combination of the aminoglycosides with natural products can be an alternative to minimize the side effects of this class of antibiotics, since the association leads to a synergistic effect significantly reducing the MIC of these drugs, decreasing the dose needed for therapeutic usage.

Many *β*-lactam antibiotics can penetrate Gram-negative bacteria via protein channels present in the outer membrane. Through these channels, the drug can reach its receptor on the cell wall and exert its bactericidal action [30]. Although extracts have in its constitution secondary metabolites such as tannins and flavonoids, which are synthesized by plants in response to microbial infections [31, 32], modifying the cell wall or disrupting the bacterial cell membrane [33, 34]. However, Gram-negative strains were not susceptible to association between the extracts and the antibiotics (Table 7). This fact can be explained by other resistance mechanisms present in these bacteria as efflux pump, production of enzymes that cleave the beta-lactam ring (*β*-lactamases), changes in PBP, among other [35, 36].

A similar fact occurs with the combination of natural products with beta-lactams against the Gram-positive strains. No modulatory effect was observed against these strains, with exception to the combination with ampicillin, which resulted in an antagonism with the MIC enhancing from 128 to 512 *μ*g/mL (Table 7). 

## 4. Conclusion

 The present results indicate that ethanolic extracts of *A. cearensis* and *A. macrocarpa* are an alternative source of natural products with antibacterial action, due to the presence of several antibacterial, which can be responsible for the observed modulatory effects, indicating the possibility of using natural products combined with aminoglycosides to increase the antimicrobial potential of these drugs against multiresistant microorganisms.

## Figures and Tables

**Table 1:** Origin of the bacterial strains and profile of resistance to antibiotics.

Bacteria	Origin	Profile of resistance
*Escherichia coli *27	Surgical wound	Ast, Ax, Amp, Ami, Amox, Ca, Cfc, Cf,
		Caz, Cip, Chlo, Im, Kan, Szt, Tet, Tob
*Escherichia coli *ATCC10536	ATCC	—
*Staphylococcus aureus *358	Surgical wound	Oxa, Gen, Tob, Ami, Kan, Neo, Para,
		But, Sis, Net
*Staphylococcus aureus *ATCC25923	ATCC	—

Ast: Aztreonam; Ax: Amoxicillin; Amp: Ampicillin; Ami: Amikacin; Amox: Amoxicillin; Ca: Cefadroxil; Cfc: Cefaclor; Cf: Cefalotin; Caz: Ceftazidime; Cip: Ciprofloxacin; Chlo: Chloramphenicol; Im: Imipenem; Kan: Kanamycin; Szt: Sulfametim; Tet: Tetracycline; Tob: Tobramycin; Oxa: Oxacillin; Gen: Gentamicin; Neo: Neomycin; Para: Paramomycin; But: Butirosin; Sis: Sisomicin; Net: Netilmicin; (—): sensitivity. ATCC: american type culture collection.

**Table 2:** Botanical families, species, and number of the title of the plants used in this study.

Family	Species	Number HCDAL	Herbarium
Leguminosae	*Amburana cearensis *	5545	Vale do São Francisco-UNIVASF
Fabaceae	*Anadenanthera macrocarpa *	6490	Dárdano Andrade Lima-URCA

**Table 3:** Dry weight and yield of ethanolic extracts (g).

Biological species	Solvent	Leaves (mass)	Extract gross (yield)
*Anadenanthera macrocarpa *	Ethanol (EEAM)	50 g	9,24%
*Amburana cearensis *	Ethanol (EEAC)	50 g	8,15%

EEAM: ethanolic extract of *Anadenanthera macrocarpa*; EEAC: ethanolic extract of *Amburana cearensis*.

**Table 4:** Phytochemical prospecting of the ethanolic extracts of *Anadenanthera macrocarpa *and *Amburana cearensis. *

Metabolites															
Extracts	1	2	3	4	5	6	7	8	9	10	11	12	13	14	15

EEAC	−	+	−	+	+	+	+	+	+	+	+	+	+	+	+
EEAM	−	+	−	+	+	+	+	+	+	+	+	+	+	+	−

1: phenols; 2: tannin pyrogallates; 3: tannin phlobaphenes; 4: anthocyanins; 5: anthocyanidins; 6: flavones; 7: flavonols; 8: xanthones; 9: chalcones; 10: aurones; 11: flavonols; 12: leucoanthocyanidins; 13: catechins; 14: flavonones; 15: alkaloids; (+): presence; (−): absence. EEAC: etanolic extract of *Amburana cearensis; *EEAM: Ethanolic Extract of *Anadenanthera macrocarpa.*

**Table 5:** Minimal inhibitory concentration (MIC) of the ethanolic extracts of *Anadenanthera macrocarpa* and of *Amburana cearensis* (*μ*g/mL).

Extracts and antimicrobials	EC 27	EC-ATCC 10536	AS 358	SA-ATCC 25923
EEAM	≥1024	≥1024	≥1024	≥1024
EEAC	≥1024	≥1024	≥1024	512

EEAM: ethanolic extract of *Anadenanthera macrocarpa*; EEAC: ethanolic extract of *Amburana cearensis*. EC: *Escherichia coli*, SA: *Staphylococcus aureus*.

**Table 6:** MIC of the aminoglycosides in the presence and absence of ethanolic extracts of *A.cearensis* and *A. macrocarpa* at a concentration 128 *μ*g/mL.

	EC 27			SA 358		
Antibiotics	MIC	MIC combined		MIC	MIC combined	
		EEAC	EEAM		EEAC	EEAM
Gentamicin	64	4	4	16	16	4
Amikacin	128	8	4	64	64	16

EEAC: ethanolic extract of *Amburana cearensis*; EEAM: ethanolic extract of *Anadenanthera macrocarpa*. EC: *Escherichia coli*, SA: *Staphylococcus aureus*.

**Table 7:** Minimal inhibitory concentration (MIC) of beta-lactam in the presence and absence of ethanolic extracts of *Amburana cearensis* and *Anadenanthera macrocarpa* and a concentration of MIC/8 (128 *μ*g/mL).

	EC 27			SA 358		
Antibiotics	MIC	MIC combined		MIC	MIC combined	
		EEAC	EEAM		EEAC	EEAM
Benzetacil	≥1024	≥1024	≥1024	512	512	512
Cephalothin	16	16	16	≤0.5	≤0.5	≤0.5
Ampicillin	≥1024	≥1024	≥1024	128	512	512
Oxacillin	≥1024	≥1024	≥1024	≤0.5	≤0.5	≤0.5

EEAC: ethanolic extract of *Amburana cearensis*; EEAM: ethanolic extract of *Anadenanthera macrocarpa*. EC: *Escherichia coli*, SA: *Staphylococcus aureus*.
